# Genetic mapping of a new heart rate QTL on chromosome 8 of spontaneously hypertensive rats

**DOI:** 10.1186/1471-2350-8-17

**Published:** 2007-04-09

**Authors:** Gustavo JJ Silva, Alexandre C Pereira, Eduardo M Krieger, José E Krieger

**Affiliations:** 1 Department of Medicine-LIM13, Heart Institute (InCor), University of São Paulo Medical School, Av. Dr. Enéas de Carvalho Aguiar, 44, 10o andar, 05403-000, São Paulo, SP, Brazil; 2 Laboratório de Genética e Cardiologia Molecular, Instituto do Coração (InCor) da Faculdade de Medicina da Universidade de São Paulo, Brazil, Av. Dr. Enéas de Carvalho Aguiar, 44 São Paulo, Brazil

## Abstract

**Background:**

Tachycardia is commonly observed in hypertensive patients, predominantly mediated by regulatory mechanisms integrated within the autonomic nervous system. The genetic loci and genes associated with increased heart rate in hypertension, however, have not yet been identified.

**Methods:**

An F2 intercross of Spontaneously Hypertensive Rats (SHR) × Brown Norway (BN) linkage analysis of quantitative trait loci mapping was utilized to identify candidate genes associated with an increased heart rate in arterial hypertension.

**Results:**

Basal heart rate in SHR was higher compared to that of normotensive BN rats (365 ± 3 vs. 314 ± 6 bpm, p < 0.05 for SHR and BN, respectively). A total genome scan identified one quantitative trait locus in a 6.78 cM interval on rat chromosome 8 (8q22–q24) that was responsible for elevated heart rate. This interval contained 241 genes, of which 65 are known genes.

**Conclusion:**

Our data suggest that an influential genetic region located on the rat chromosome 8 contributes to the regulation of heart rate. Candidate genes that have previously been associated with tachycardia and/or hypertension were found within this QTL, strengthening our hypothesis that these genes are, potentially, associated with the increase in heart rate in a hypertension rat model.

## Background

Heart rate is primarily determined by regulatory mechanisms that are integrated within the autonomic nervous system. In arterial hypertension, an increased heart rate is commonly observed as a compensatory response to decreased stroke volume (due to either a diastolic or systolic myocardial dysfunction) [1,2]. Although an increased basal heart rate, accompanying myocardial dysfunction, might represent a secondary phenomenon in established hypertension, an early increase in heart rate may contribute directly to the pathogenesis of the disease [3-6]. In fact, tachycardia in arterial hypertension seems to be a heritable trait and independent of reflex responses [7]. Few studies, however, have focused on the identification of the genetic basis of tachycardia related to hypertension. A total genome scan to identify quantitative trait loci (QTL) provides a powerful tool to determine the chromosomal location of alleles and polygenes that contribute to complex diseases, such as autoimmune disorders [8,9], cardiovascular diseases [10,11], cancer [12,13], infectious diseases [14] and drug addiction [15,16].

Even though QTLs for complex diseases have been identified, little progress has been made towards the identification of the genes related to complex traits such as increased heart rate associated with hypertension. Recently, Jaworski et al. [17] reported a QTL in rat chromosome 2, centered around the D2Rat61/62 markers, that is related to the air-puff stimulus-induced bradycardia response. In agreement with Jaworski, Alemayehu et al. [18] described a heart rate QTL involved in bradycardia, induced by salt-load diet, on rat chromosome 2. We previously mapped five QTL's (two on chromosome 2, and one each on chromosomes 4, 8 and 16) involved in blood pressure variation in an F2 population of an intercross between Brown Norway (BN) and Spontaneously hypertensive rats (SHR) [19]. In this study, we used linkage analysis for QTL mapping and identification of candidate genes associated with basal levels of heart rate in the progenies of an F2 rat intercross between the BN and SHR strains.

## Methods

### Parental rat strain crosses and F2 phenotyping

To construct a detailed linkage map of all chromosomes and determine the genetic traits for tachycardia associated with hypertension we analyzed an intercross (F2) progeny (N = 188 rats) of the BN and the SHR strains, and normotensive (BN, N = 4) and hypertensive (SHR, N = 23) rats as parental strains. All animals were genotyped for 179 polymorphic markers distributed along the rat genome, according to a standard protocol [20]. Basal heart rate levels of the F2 progeny were obtained from a previously described phenotype database [19], and data from SHR and BN strains (Medical School, University of São Paulo) were evaluated in male 12–14 week old animals. Briefly, an arterial polyethylene catheter (PE-10, 0.28 mm ID, 0.61 mm OD, Biocorp Australia, Huntingdale, Victoria, Australia) was implanted under anesthesia (Pentobarbitone sodium, 30 mg/kg, i.p.) into the abdominal aorta through the left femoral artery at least 24 hours before measurements. Blood pressure was measured in non-anesthetized, freely moving rats, on a beat-to-beat basis at a 1.0 KHz of sample rate (DataQ Instruments, Inc. Ohio, USA) by connecting the arterial cannula to a pressure transducer (Statham P_23_Dd, Hato Rey, Puerto Rico). The pulse pressure signal from the transducer was fed to an amplifier (GPA-4 model 2, Stemtech, Inc., Wood Dale, IL, USA) and then to a 10-bit analog-to-digital converter (DataQ Instruments, Inc., Akron, OH, USA). Basal heart rate was derived from pulsatile blood pressure. All animal experimental procedures were followed in accordance to the guidelines for animal care and use of laboratory of the University of São Paulo, Brazil and the protocol approved by designated ethics committee.

### Genetic marker map

The 188 animals from the F2 progeny were previously genotyped for 179 genetic markers distributed along the whole genome [19]. Although no new genotypes were incorporated for this study, we have recently re-accessed the genetic distances of each genotyped marker [21] through sequential consultation of different internet databases [22-24].

### Quantitative trait loci (QTL) mapping

To assess the contribution of a putative QTL to the heart rate trait, we used the composite interval mapping method [25-28] in the F2 population (described above) using the Windows QTL Cartographer [29]. We then determined the significance of putative QTL's from the interval mapping scan based on a likelihood ratio (LR) threshold and considered 11.5 as suggestive linkage.

### Criteria for candidate gene identification

Genes presented on the mapped heart rate QTL were listed by consulting the public database of the rat genome [30]. The selection of candidate genes proceeded according to the following criteria. Firstly, the known genes presented in the mapped QTL were listed; secondly, the known genes were submitted to gene ontology analysis (described below) for refinement of selection; and finally, genes were considered as candidate only if presented in a heart rate related phenotype, confirmed by citations on the NCBI PubMed browser [31].

Annotation of functional classes was carried out using information provided by the Gene Ontology database [32], accessed by NCBI's public repository [33]. The known genes found in the QTL were classified in specific function, process, or component classes through the GO terms assignation. The genes belonging to the same class were grouped and listed, and the candidate genes were elected from the most frequent gene ontology terms or according to gene ontology terms that are directly related to the heart rate trait.

### Statistical analysis

Kolmogorov-Smirnov test (with Lilliefors' correction) was used to test data for normality of the estimated underlying population using SigmaStat 3.11 (Systat Software, Inc., San Jose, CA, USA). Normality was assumed for all parametric tests and regression procedures. All phenotype measurements (blood pressure and heart rate) were compared between each parental strain (SHR × BN) using the Student *t *test. Correlation between blood pressure and heart rate values was performed calculating the Pearson r correlation coefficient. Statistical significance was establish according to the P value (p < 0.05) for a two-tailed distribution. All data are reported as mean ± SEM.

## Results

### Hemodynamic correlations

As expected, inbred parental strains had different levels of basal blood pressure and heart rate (Table 1). Systolic (188 ± 2 vs. 127 ± 5 mm Hg), diastolic (138 ± 2 vs. 91 ± 4 mm Hg), and mean (162 ± 2 vs. 109 ± 5 mm Hg) blood pressure measurements were significantly increased in SHR compared to the normotensive BN strain. Moreover, the SHR strain presented an increase in heart rate levels (365 ± 3 vs. 314 ± 6 bpm, p < 0.0001) compared to BN. Although heart rate significantly correlated (p = 0.0069 for systolic blood pressure) with blood pressure levels (Table 2), the R squared value suggested that these variables are poorly correlated only (0.0335 for systolic blood pressure). Thus, we did not adjust heart rate values for systolic or diastolic blood pressure traits before QTL mapping analysis.

**Table 1:** Systolic (SBP), diastolic (DBP), and mean (MBP) blood pressure, and heart rate (HR) in normotensive (Brown Norway) and hypertensive (SHR) rat strains.

	**Brown Norway** **(N = 04)**	**SHR** **(N = 23)**	***p***
**SBP (mm Hg)**	127 ± 5	188 ± 2	< 0.0001
**DBP (mm Hg)**	91 ± 4	138 ± 2	< 0.0001
**MBP (mm Hg)**	109 ± 5	162 ± 2	< 0.0001
**HR (bpm)**	314 ± 6	365 ± 3	< 0.0001

**Table 2:** The correlation between heart rate and mean (MBP), diastolic (DBP) and systolic (SBP) blood pressures obtained in an F2 (SHR × Brown Norway) population.

	**R-squared**	**Pearson r**	**Confidence interval**	***P *(two-tailed)**
**vs. MBP**	0.0352	-0.1877	-0.3131 to -0.05587	0.0055
**vs. DBP**	0.0335	-0.1830	-0.3087 to -0.05098	0.0069
**vs. SBP**	0.0335	-0.1830	-0.3087 to -0.05105	0.0069

### QTL mapping

**Figure 1:**
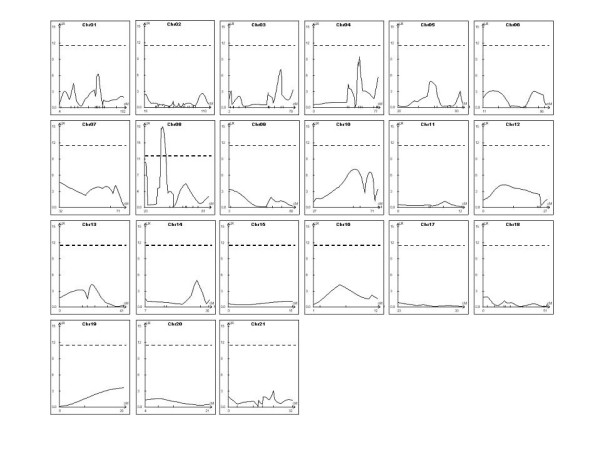
Likelihood ratio (LR) statistics for basal heart rate trait for all chromosomes in 188 rats from an F2 intercross (SHR × Brown Norway) population. Each panel represents the chromosome number with the genetic length of the chromosome (cM) on the abscissa, and the horizontal dashed line denotes an LR score of 11.5 as the threshold for suggestive linkage.

**Figure 2:**
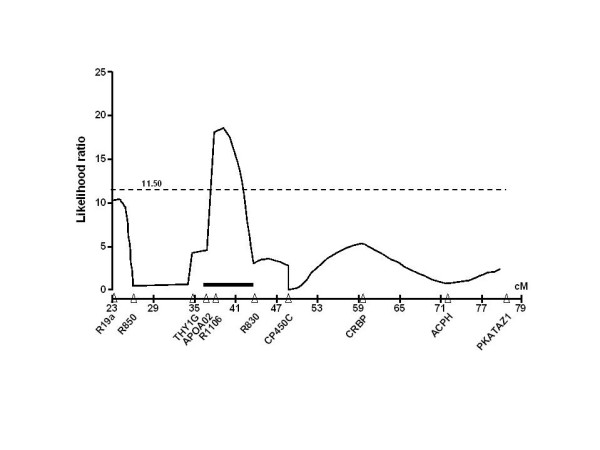
Rat chromosome 8 heart rate QTL in 188 rats from an F2 intercross (SHR × Brown Norway) population. The horizontal dashed line denotes the likelihood ratio (LR) of 11.5 as the threshold for suggestive linkage. See Table 3 for details of markers delimiting the QTL and the effects on heart rate, according to genotype.

The linkage signals for the composite interval mapping based on 179 markers along the 21 rat chromosomes (Figure 1) revealed the presence of a putative QTL responsible for an increase in heart rate in chromosome 8 (Figure 1). Figure 2 shows, in detail, the 6.78 cM heart rate QTL mapped on rat chromosome 8 (8q22–q24), flanked by APOA02 and R830 markers. In addition, the likelihood ratio statistics for the higher linkage signal of the putative QTL in chromosome 8 was 18.67. The estimated effects on heart rate are -27.3 bpm for the additive and 5.3 bpm for the dominant terms. Table 3 summarizes the allelic effects on heart rate levels for the APOA02, R1106, and R830 markers in homozygotes for either BN (BN/BN) and SHR (SHR/SHR), and heterozygotes (BN/SHR) in the F2 intercross (SHR × BN) population. Although no significant difference was observed in heart rate level for the APOA02 (p = 0.7614) and the R830 (p = 0.8963) markers, an allelic effect was observed for the R1106 marker (p = 0.0306). SHR homozygotes presented significantly lower heart rate values compared to BN homozygotes (377 ± 6 vs. 370 ± 3 vs. 354 ± 8 bpm, for BN/BN, BN/SHR, and SHR/SHR, respectively).

**Table 3:** Heart rate distributions, according to the genotype (BN/SHR, BN/SHR, or SHR/SHR), for the APOA02, R1106, and R830 markers that flanked the heart rate QTL mapped in a 188 F2 (SHR × Brown Norway) population.

**Markers**	**cM**	**Heart rate by genotype**			**One-way ANOVA (P)**	**Heart rate effect (bpm)**
				
		**BN/BN**	**BN/SHR**	**SHR/SHR**		
**APOA02**	36.87	370 ± 8 (N = 43)	367 ± 3 (N = 91)	364 ± 4 (N = 46)	0.7614	5.81
**R1106**	38.10	377 ± 6 (N = 29)	370 ± 3 (N = 92)	354 ± 8* (N = 37)	0.0306	22.87
**R830**	43.65	368 ± 8 (N = 41)	368 ± 3 (N = 91)	365 ± 4 (N = 43)	0.8963	3.20

*p < 0.05 when compared to BN/BN.

Note that heart rate effect (bpm) represents the heart rate difference between BN/BN and SHR/SHR genotypes.

### Candidate gene identification

The 6.78 cM heart rate QTL segment mapped on rat chromosome 8 harbours 241 genes. When only known genes were considered (Table 4), 176 genes were initially excluded. Gene ontology analysis then revealed that most of the 65 known genes were related to "metal ion binding", "protein binding", or "synaptic transmission" functions, and some of these presented "receptor/transporter/channel activity", some of which have an ion channel activity (Chrna5, Chrna3, Chrnb4, Htr3a, Htr3b, Fxyd2, and Hcn4). After gene ontology analysis, a further 48 genes were excluded, and 17 candidate genes remained. Ten of the genes investigated (Bace1, Timm8b, Ncam1, Slc37a4, Cul5, Fxyd2, Fxyd6, Ptpn9, Scamp2 and Scamp5) did not display any association with the heart rate trait and were eliminated. Candidate genes that were associated with the heart rate trait are described in detail in Table 5: Nicotinic acetylcholine receptor subtypes α3 (Chrna3), α5 (Chrna5), and β4 (Chrnb4); hyperpolarization-activated channel (Hcn4); 5-hydroxytryptamine (serotonin) receptor 3a (Htr3a) and 3b (Htr3b); and sodium channel (Scn2).

**Table 4:** Known genes present on the heart rate QTL mapped on SHR chromosome 8, and classified according to Gene Ontology (GO) terms.

**GO Terms**	**Gene ID**	**Description**
Axon	Bace1	beta-site APP cleaving enzyme 1
		
Metabolism	Mpi_mapped	mannose phosphate isomerase (mapped)
	Idh3a	isocitrate dehydrogenase 3 (NAD+) alpha
	Man2c1	mannosidase, alpha, class 2C, member 1
	Pkm2	pyruvate kinase, muscle
		
Metal Ion Binding	Pts	6-pyruvoyl-tetrahydropterin synthase
	Fdx1	ferredoxin 1
	Cyp19	cytochrome P450, subfamily 19
	Cyp1a2	cytochrome P450, 1a2
	Cyp11a	cytochrome P450, subfamily 11A
	Ireb2	iron-regulatory protein 2
	Isl2	insulin related protein 2
	Cox5a	cytochrome c oxidase, subunit Va
		
Nucleus	Cryab	crystallin, alpha B
	Atm_mapped	ataxia telangiectasia mutated homolog (human) (mapped)
		
Protein Folding/Processing	Hspb2	heat shock 27 kD protein 2
	Hyou1	hypoxia up-regulated 1
	Pcsk7	proprotein convertase subtilisin/kexin, type 7
		
Protein Binding	Mcam	l-gicerin
	Thy1	thymus cell antigen 1, theta
	Usp2	ubiquitin specific protease 2
	Zbtb16	zinc finger and BTB domain containing 16
	Cul5	vasopressin-activated calcium-mobilizing receptor protein
	Rcn2	reticulocalbin 2
	Cd3d	CD3 antigen delta polypeptide
	Tagln	transgelin
		
Receptor/Transporter/Channel Activity	Blr1	Burkitt lymphoma receptor 1
	Il10ra	interleukin 10 receptor, alpha
	Neo1	neogenin
	Slc37a4	solute carrier family 37 (glycerol-6-phosphate transporter), member 4
	Fxyd2	FXYD domain-containing ion transport regulator 2
	Fxyd6	FXYD domain-containing ion transport regulator 6
	Hcn4	hyperpolarization-activated, cyclic nucleotide-gated K+ 4
	Timm8b	translocase of inner mitochondrial membrane 8 homolog b (yeast)
	Scamp2	secretory carrier membrane protein 2
	Scamp5	secretory carrier membrane protein 5
		
Synaptic Transmission	Chrna5	cholinergic receptor, nicotinic, alpha polypeptide 5
	Chrna3	cholinergic receptor, nicotinic, alpha polypeptide 3
	Chrnb4	cholinergic receptor, nicotinic, beta polypeptide 4
	Scn2b	sodium channel, voltage-gated, type II, beta polypeptide
	Htr3b	5-hydroxytryptamine (serotonin) receptor 3b
	Htr3a	5-hydroxytryptamine (serotonin) receptor 3a
	Ncam1	neural cell adhesion molecule 1
		
Transferase Activity	Hmbs	hydroxymethylbilane synthase
	Dlat	dihydrolipoamide acetyltransferase
	Acat1	acetyl-coenzyme A acetyltransferase 1
	Clk3	CDC-like kinase 3
		
Other Terms	Rps25	ribosomal protein s25
	Treh	trehalase
	Pafah1b2	platelet-activating factor acetylhydrolase alpha 2 subunit (PAF-AH alpha 2)
	Apoa5	apolipoprotein A-V
	Tmprss5	transmembrane protease, serine 5 (spinesin)
	Il18	interleukin 18
	Crabp1_mapped	cellular retinoic acid binding protein I (mapped)
	Psma4	proteasome (prosome, macropain) subunit, alpha type 4
	Cspg4	membrane-spanning proteoglycan NG2
	Ptpn9	protein tyrosine phosphatase, non-receptor type 9
	Sdfr1	stromal cell derived factor receptor 1
	Hexa	hexosaminidase A
	Myo9a	myosin IXA
	Ube4a	ubiquitin conjugation factor E4 A
	Cd276	CD276 antigen
		
No Terms	Phldb1	pleckstrin homology-like domain, family B, member 1
	Crgl2	olfactomedin-related protein
	Znf291	KIAA1454-like protein

**Table 5:** Associated phenotypes described for 13 candidate genes present on the heart rate QTL, mapped on SHR chromosome 8.

**Gene**	**Name**	**Associated phenotype**	**Reference**
Chrna3 Chrna5 Chrnb4	Cholinergic receptor, nicotinic, alpha polypeptide 3 and 5, and beta polypeptide 4	- Autonomic nervous system influence on heart rate; - Alzheimer's disease; - Autosomal dominant nocturnal frontal lobe epilepsy.	Bonati et al., 2000 [46] Bonati et al., 2002 [47] Duga et al., 2001 [43] Kawamata et al., 2002 [48] Lev-Lehman et al., 2001 [44] Liang Y 2005 [49] Rassadi, S 2005 [50] Rempel et al., 1998 [45] Wang et al., 2002 [51] Wang et al., 2003 [52]
Hcn4	Hyperpolarization-activated, cyclic nucleotide – gated K^+ ^4	- Familial sinus bradycardia; - Generation of pacemaker potentials in the sinoatrial node; - Cardiac arrhythmia.	Leoni et al., 2005 [53] Milanesi et al., 2006 [42] Schulze-Bahr et al., 2003 [54] Stieber et al., 2006 [55] Stieber et al., 2003 [40] Ueda et al., 2004 [41]
Htr3a Htr3b	5-hydroxytryptamine (serotonin) receptor 3a and 3b	- Modulatory role in the reflex control of the heart rate by cardiopulmonary receptors; - Nausea induced by paroxetine; - Fibromyalgia; - Numerous human neuropsychiatric disorders (female major depression, bipolar affective and schizophrenic).	Aviado et al., 2001 [56] Frank et al., 2004 [57] Yamada et al., 2006 [58] Iidaka et al., 2005 [59] Niesler et al., 2001 [60] Sevoz et al., 1997 [61] Sugai et al., 2006 [62] Thoren, 1979 [63] Whalen et al., 2000 [64]
Scn2b	Sodium channel, voltage-gated, type II, beta polypeptide	- Essential hypertension; - Liddle's syndrome; - Idiopathic generalized epilepsy; - Neuropathic pain; - Cell adhesion and migration; - Chronic Heart Failure.	Baker et al., 1998 [65] Botero-Velez et al., 1994 [66] Dong et al., 2002 [67] Haug et al., 2000 [68] Kim et al., 2005 [69] Pegoraro RJ et al., 2004 [70] Pertin et al., 2005 [71] Schild et al., 1996 [72] Snyder et al., 1995 [73] Su et al., 1996 [74] Rayner et al., 2003 [75] Zicha S et al., 2004 [76]

## Discussion and conclusion

A number of reports studying hypertensive patients have demonstrated an increased basal heart rate accompanying myocardial dysfunction [1-6]. Furthermore, a positive association between heart rate and all-cause mortality or cardiovascular mortality has been observed [5,6]. As such, it is important to elucidate the genetic factors that lead to increased heart rate levels. Based on the observation of an increased basal heart rate in the SHR strain, and using a powerful genomic exploratory approach, we mapped a new QTL on the rat chromosome 8 that is associated with basal levels of heart rate in SHR and identified several genes that could potentially be responsible for heart rate modulation.

We, herein, show that the parental SHR strain displayed increased heart rate values compared to normotensive BN rats. Moreover, in the F2 intercross animals, the allelic effects on heart rate levels for the R1106 marker suggest a complex interaction of alleles. The average heart rate values were lower in the homozygote SHR allele compared with the homozygote BN allele F2 animals. Furthermore, we observed a negative allelic effect for the R1106 marker (-22.9 bpm) that is concordant with the additive effect obtained by composite interval mapping (-27.3 bpm). These data suggest that the putative QTL, mapped on rat chromosome 8, harbours gene(s) that negatively influence the heart rate levels. Moreover, it should be taken into consideration that the algorithm of the composite interval mapping method considers the influence of other chromosomal regions or genes on the effect of the mapped QTL.

Total genome scan analysis has contributed to the discovery of several QTLs associated with different phenotypes in complex diseases. However, although relative success has been achieved in the identification of monogenic diseases [34,35], limited progress has been made in the identification and confirmation of candidate genes in complex diseases. Different chromosomal regions, associated with heart rate-phenotype traits, have been mapped in both patients and experimental animals [7,17,18,36,37]. Kreutz et al. [36] mapped a QTL on rat chromosome 3 that was associated with both basal heart rate and salt-induced changes in heart rate in an F2 intercross (SHRSP × WKY) population. Jaworski et al. [17] identified independent chromosomal regions related to bradycardia in response to mild stress (airpuff startle). Alemayehu et al. [18] identified one putative heart rate locus on rat chromosome 2 and confirmed its presence in different congenic strains after the substitution of the mapped interval from the SHR for the allelic region of a normotensive strain. Interestingly, Kren et al[38] reported a blood pressure QTL on chromosome 8 which maps nearby the heart rate-related QTL reported in the present study. However, a congenic SHR-Lx strain harbouring this particular region showed only changes in basal blood pressure and cardiac mass with no heart rate effects. Therefore, it is less likely that the same gene(s) are influencing the effects on heart rate here described. In a mouse model, Sugiyama et al. [37] conducted a QTL mapping analysis in an F2 progeny (CBA/CaJ × BALB/cJ) and identified 3 loci associated with heart rate, two on the mouse chromosome 2, and one on chromosome 15. We, herein, mapped a new QTL located in chromosome 8 and associated with a negative effect on heart rate in the SHR. Kahn and collaborators [39] found the rat chromosome 2 to be involved in heart rate, but were not able to confirm their findings in a congenic strain after the substitution of the mapped interval from the SHR for the allelic region of a normotensive BN strain. With regard to the rat chromosome 8, we previously mapped a putative QTL [19], flanked by R19 and R850 markers, associated with salt-induced changes in blood pressure that is located relatively close to the QTL, demonstrated herein, that is associated with heart rate trait.

In humans, loci associated with the autonomic nervous system's control of the heart rate have also been mapped [7]. Singh et al. [7] investigated 725 patients from the Framingham Heart Study and mapped 2 QTLs (chromosomes 2 and 15) associated with the frequency domain of heart rate variability indexes. Although the authors [7] have not mapped a QTL associated specifically with basal heart rate, they proposed that the region contains genes that share function for both basal heart rate and heart rate variability traits. Interestingly, the QTL mapped on chromosome 15 in humans is a syntenic region compared with the interval we mapped for basal heart rate traits in the rat chromosome 8.

In the present investigation, interval mapping revealed 17 genes that are potentially implicated in basal heart rate in the SHR. Among these candidate genes, just 4 families of genes were identified that maintained the association with the heart rate phenotype after the use of certain selection criteria; these genes were Scn2b (Sodium channel type II, beta polypeptide); Htr3a and Htr3b (5-Hydroxytryptamine receptors type 3); Hcn4 (Hyperpolarization-activated, cyclic nucleotide-gated K^+ ^type 4); Chrna3, Chrna5, and Chrnb4 (Neuronal nicotinic acetylcholine receptor, alpha polypeptide 3 and 5, and beta polypeptide 4). Interestingly, association studies and genetic engineered mice data support the involvement of some of the heart rate candidate genes in arterial hypertension.

Knockout mice for the hyperpolarization-activated, cyclic nucleotide – gated K^+ ^4 (HCN4) gene have cardiac cells with "mature" pacemaker potentials, demonstrating that this gene that encodes a hyperpolarization-activated channel is essential for the proper generation of pacemaker potentials in the sinoatrial node [40]. In patients, a missense D553N mutation in the HCN4 gene was associated with sinus node dysfunction, QT prolongation in the electrocardiogram, polymorphic ventricular tachycardia, and syncope [41]. In addition, the presence of the S672R mutation in the coding region of the HCN4 gene, mimics a mild vagal stimulation in the sinoatrial node, slowing the heart rate by decreasing the inward diastolic current [42]. These data suggest that loss of function of the HCN4 gene is associated with sinus nodal dysfunction.

The neuronal nicotinic acetylcholine receptor (Chrn) subtypes α3, α5, and β4 are members of a ligand-gated ion channel family that affect sodium and potassium transients. Knockout mice strains lacking the genes encoding the subtypes α5 and β4 of the nicotinic receptors showed cardiac autonomic dysfunction, affecting the heart rate response to vagal electrical stimulation. In humans, although intragenic polymorphisms on the nicotinic receptor subunits have been identified [43-45], these gene mutations have not yet been associated with cardiovascular phenotypes.

One potential study limitation is the number of genetic markers used in the genome-mapping experiment. Although a large number of F2 analyses have been reported, genes responsible for SHR hypertension and/or heart rate related phenotypes have not been yet identified. One of the possible explanations for this fact is the small number of markers genotyped in these studies. We used 179 informative markers in our mapping experiment using an intercross between the SHR and BN strains. Although genetic distances and locations of these markers were updated before conducting our analysis, it is possible that QTLs important for the phenotype studied remain unidentified.

Our data suggest that genetic markers on the rat chromosome 8 contribute to heart rate control in experimental hypertension. The recognition of genetic determinants of heart rate may help gain insights into the pathophysiology of the autonomic nervous system and offer clues as to its modulation in hypertension. Further studies will be necessary to identify the specific gene(s) in chromosome 8 that are involved in hypertension-related tachychardia and characterize the molecular mechanisms underlying the genetic influence on heart rate in hypertensive rats.

## Competing interests

The author(s) declare that they have no competing interests.

## Authors' contributions

GJJS carried out the molecular genetic studies, participated in the sequence alignment and drafted the manuscript. ACP carried out the molecular genetic studies and drafted the manuscript. EMK conceived of the study, and participated in its design and coordination. JEK conceived of the study, and participated in its design and coordination. All authors read and approved the final manuscript.

## Pre-publication history

The pre-publication history for this paper can be accessed here:


